# Overlapping of clinical symptoms between schizophrenia and bipolar disorder

**DOI:** 10.1192/j.eurpsy.2022.919

**Published:** 2022-09-01

**Authors:** A. Tahirovic, M. Arnautović Tahirović, M. Muhic, H. Sikira, G. Sulejmanpasic, L. Haracic Curovac, A. Cesir, A. Memic Serdarevic

**Affiliations:** 1 Clinical Centre University of Sarajevo, Psychiatric Clinic, Sarajevo, Bosnia and Herzegovina; 2 Psychiatric Hospital of Canton Sarajevo, Clinical Psychiatry, Sarajevo, Bosnia and Herzegovina

**Keywords:** schizophrénia, YMRS, PANSS, Bipolar Affective Disorder

## Abstract

**Introduction:**

Schizophrenia (SCH) and bipolar affective disorder (BP) are complex disorders that overlapping both in their clinical symptoms and certain familiar characteristics. They share some common characteristcs but there are also key differences. The frequency of overlapping symptoms between these diseases could give us more information about the current validity of the diagnosis based on existing diagnostic criteria. Similarities within and between these two disorders in the future, can possibly redefine greater reliability of diagnosis.

**Objectives:**

The aim of the study was to investigate the frequency of overlapping symptoms between BP and SCH.

**Methods:**

The sample included 159 patients diagnosed with SCH and 61 with BP who were followed over a two year period. The research was conducted at the UCCS Psychiatric Clinic. Assessment of clinical symptoms and diagnosis were performed using a structured clinical interview (SCID I), a list of operationalized criteria (OPSCRIT), a scale for the assessment of positive and negative symptoms (PANSS), a scale for the assessment of manic symptoms (YMRS).

**Results:**

The overall PANSS score was significantly higher in patients with SCH compared to patients with BP, but on the general psychopatology there are no significant differences betwen SCH and BP. Symptoms of mania are significantly more pronounced in patients with BP compared to those with SCH.

**Conclusions:**

Our results of overlapping of individual symptoms between SCH and BP can speak infavor of the theory of disease continuum. And can also help us in understanding symptoms and guide us to develop optimal treatment strategies.

**Disclosure:**

No significant relationships.

